# Corpus Christi Meeting of Association of Texas Health Officers

**Published:** 1908-09

**Authors:** L. B. Bibb

**Affiliations:** Austin, Texas


### TEXAS MEDICAL JOURNAL.

Established July, 1885.

F. E. DANIEL, M. D., - - - - Editor, Publisher and Proprietor.

PUBLISHED MONTHLY.—SUBSCRIPTION $1.00 A YEAR.

VOL. XXIV. AUSTIN, SEPTEMBER, 1908. No. 3.

The publisher is not responsible for the views of contributors.

### Department of Public Health.

### Corpus Christi Meeting of Association of Texas Health Officers.

BY L. B. BIBB, M. D., AUSTIN, TEXAS.

(Continued.)

Williamson county, represented by Dr. E. M. Thomas, was next heard from as follows:

Williamson county has made some sanitary improvements on jail and over the county generally. Some improvements are being made on slaughter houses, beef markets, etc. Most of the towns have scavengers. No sanitary forces that I know of have been organized.

PURE FOOD.

No milk and food inspection.

QUARANTINE.

Since January 1, 1908, have had about fifty cases of smallpox, one death; scarlet fever, only six reported, no deaths; diphtheria, six reported, one death; tuberculosis, four reported, one death; epidemic dysentery, not reported by physicians, but we have an epidemic of it with numerous cases, and one death that I have heard of. Our county only quarantines smallpox. The physicians are co-operating with me, and report all cases of this disease to me, and the commissioners court are supporting me in this one disease, and have passed quarantine resolutions and authorize me to take control of any cases that may come to my knowledge. Resolutions have been passed by most of our schools requiring vaccination for entrance, but it is not enforced. Very few physicians report tuberculosis, and when they do I do not take any control in this disease whatever; no perceptible increase in tuberculosis. Our people are becoming interested in the necessity of mosquito extermination, and most of them are screening their houses, and are interested in better drainage. I am opposed to quarantine in the case that you relate. I think our people, as a rule, would listen to reason when assured by me that the State Health Officer and I were doing all that was necessary to prevent an epidemic of any kind. I have read the sanitary code, and I think our people are reasonable in anything for their safety.

VITAL STATISTICS.

About 85 per cent of births and deaths are reported to county clerk at present, and about 85 per cent of doctors are reporting births and deaths. Midwives report better than doctors.

Ward county, represented by Dr. N. W. Gustine, was next heard from as follows:

There is some interest being taken in our county in the sanitary affairs, but owing to the high altitude and light rainfall in our section there is not the necessity for precaution that exists in other parts of the State. We have, however, made some improvements in the keeping of our court house and jail, the court having employed a regular janitor for that purpose; the school trustees have also employed a regular janitor, and both court house and jail and the public school house are being properly kept and disinfected. Our slaughter pens are situated outside of the town and in the open country and are dry and reasonably clean; our meat markets, while not kept in strict compliance with the regulations, are in a very decent and healthy condition. I, as the county health officer, have been exercising a close watch over contagious diseases, with a reasonable care for the general sanitary conditions. There has been no certain appropriation made for these things.

PURE FOOD.

We have no milk or food inspection in any of the towns of our county, and there is really no need for such.

QUARANTINE.

There have been fifteen cases of smallpox reported during the year, and there have been probably as many more not reported, all these cases have been of the very mild attenuated form and no deaths from any of these cases; there have been nine cases of scarlet fever reported this year, all cases very mild and no deaths; there has been no case of any of the other diseases mentioned or required in this report this year. The regulations on quarantine, isolation and disinfection have been strictly complied with in all cases of smallpox and scarlet fever and the physicians are all in accord and are co-operating with me. The commissioners court has authorized me to take the immediate control of all cases of contagious diseases and to employ such help as is necessary to take proper care of same, and to maintain all necessary quarantine. The public schools do not require compulsory vaccination. Physicians have not been reporting cases of tuberculosis, and, in many instances, I have failed to follow up and disinfect the house after removal. There has been only one death reported this year from tuberculosis; there is no perceptible increase in the number of cases occurring among the older residents, but there are a good number of patients that come here from other parts of the country seeking health.

Our people are very much alive to the necessity of the extermination of the mosquito, and to that end are making and fixing to make great improvements in our drainage system; at some seasons of the year we have a great number of mosquitoes, but all of the Culex variety or those not capable of carrying disease.

Were I sure that there were only one or two imported cases of yellow fever in a town or city, and further assured that the disease was recognized early and all necessary precautions were taken from the beginning, I should think quarantine of the town unnecessary. My constituents are very intelligent, and I feel that they will listen to reason from any reliable source and especially from our health officers, and will stand by me in any sane method of protection against disease.

I have not read the proposed sanitary code for unincorporated towns, but am sure our citizens will incorporate under the laws governing for sanitary purposes.

### VITAL STATISTICS.

The per cent of births and deaths reported in the county at present is approximately 75. The regular physicians are all reporting now, but the midwives are not reporting. We have a good number of Mexicans here and with their ignorance it is impossible to ever have a correct vital statistics report.

Newton county, represented by Dr. B. A. Swinney, was next heard from as follows:

Upon receipt of your proposed code of sanitary laws for the government of unincorporated towns in this State we had a mass meeting of the citizens of the town which resulted in the appointment of a committee to examine said code of laws and select such as were in their judgment applicable to conditions in our town. Said committee to report the result of their investigation with recommendations for adoption by another mass meeting of the citizens which rejected the report and are now circulating two petitions to the commissioners court, which I think will result in a defeat for incorporation, as some of our best citizens are opposing it.

There has been no improvement in sanitary conditions in court house and jail except some attention is now being paid to cleaning cuspidores in the court house. No attention is paid to clean-up days. No towns in the county have organized sanitary forces, and it is a difficult matter to get the people interested.

PURE FOOD.

No effort has been made in the county to establish a food and milk inspection service.

QUARANTINE.

There has been no leprosy, smallpox, scarlet fever, diphtheria, epidemic cerebro-spinal fever or trachoma in the county since January, 1908. There has been some typhoid fever, dysentery and tuberculosis. Two deaths reported from tuberculosis only this year. The commissioners court has given me authority to act in all cases of contagious or infectious diseases without first consulting the court.

Compulsory vaccination has not been enforced in our county as we have had no smallpox for a number of years. Physicians in the county are not reporting tuberculosis, so that it is impossible to disinfect as the laws require in case of death or removal. I think that there is an increase in tuberculosis, especially among the colored race in the last few years.

Our people can not be aroused as to the need of mosquito extermination. There is a little screening of houses done in this section and no attention at all paid to cisterns and wells as a rule; neither has there been much attention paid to drainage anywhere in the county, so I think yellow fever might give our people some trouble in this county, as we have more or less mosquitoes most of the year. I think, however, that in case of imported cases of fever early recognized and properly screened and with all other necessary precautions that our people would be willing to trust the health departments of State and county, and I should certainly not, under such conditions, favor placing on a quarantine.

I have read the proposed sanitary code for unincorporated towns, but am afraid that the people in the unincorporated towns in the county will not take kindly to it, judging from experience in home town.

### VITAL STATISTICS.

I do not think that more than 50 per cent of births and deaths are being reported to the county clerk. Some physicians in the county never report at all, others report irregularly. Midwives do not report any better than the physicians.

Refugio county, represented by Dr. J. J. Adkins, was next heard from as follows:

There is no interest taken by our people in regard to sanitary affairs whatever. There is no sanitary organization in the town or county.

### PURE FOOD.

There is no effort being made to have milk and food inspection service in our county.

### QUARANTINE.

There has been no disease to quarantine, only one of smallpox, and he stayed at home till all danger was over. One death of pulmonary tuberculosis. Since the first of January, 1908, the physicians are co-operating. The county judge and the commissioners court are willing and ready to support us in putting on quarantine when necessary. Vaccination is not compulsory in our public schools. The physicians have not been reporting tuberculosis, but I think will in the future. I did not disinfect the premises of the party that died here with tuberculosis, as I did not know that he was dead for several days after his death. I think there is an increase of tuberculosis in the past five years.

Our people are alive to the necessity of eliminating and exterminating the mosquito, but they are not doing so, and if they are interested in better drainage they don't say so. I am not in favor of placing on a quarantine against yellow fever in any town or city in the State where we are assured that the disease was recognized early and all possible precaution was taken from the beginning.

VITAL STATISTICS.

All deaths and births to the best of my knowledge have been reported for 1908, but in the past about two-thirds reported.

Robert's county, represented by Dr. M. L. Gunn, was next heard from as follows:

Great interest is being manifested along sanitary lines so far as clean-up days are concerned. No improvement on court house or jail. We have no slaughter house and no dairy. One meat market fairly well kept.

PURE FOOD.

No effort being made to have milk and food inspection service.

QUARANTINE.

No contagious diseases since January 1st, except three cases of typhoid fever and two cases of trachoma; no deaths from typhoid fever. Regulations on quarantine, isolation and disinfection have been complied with when necessary. Only one other physician in county; have had no opportunity of testing him in these matters. The commissioners court supports me in controlling all contagious diseases and have authorized me to take immediate control of these diseases. No compulsory vaccination for entering public school. There is not a case of tuberculosis in the county, so far as I know; have had only two cases in the county in the last three and a half years, and they both moved out of the county and the house was disinfected. The advisability of incorporating for sanitary purposes is under consideration at the present time.

VITAL STATISTICS.

About 75 per cent of births and deaths have been reported in the last six months. The other physician in town has failed to make his reports up to the present time. Midwives hardly ever report a case. I have never failed to report a case since the law went into effect.

San Patricio county, represented by Dr. A. P. Frick, was next heard from as follows:

In a county having a population of only about 3000 there would naturally be a few conditions calling for an elaborate report on sanitary matters. In absence of municipal organizations but little can possibly be done to organize "sanitary forces." No reports on tuberculosis have, as yet, been received. I believe that, with proper presentation, the citizens of our unincorporated towns can be interested in incorporating for sanitary purposes. As far as the town of Aransas Pass is concerned, during the last sixteen years voluntary efforts to preserve uncontaminated our excellent subterranean water supply has been a conspicuous failure. Incorporation for sanitary purposes would certainly be very helpful in many ways.

Tarrant county, represented by Dr. R. B. West, was next heard from as follows:

Our people are alive to the necessity of better sanitary conditions, and as evidence of their increasing interest the conditions are much improved in jail, court house, school house, churches, slaughter houses, dairies, meat markets, etc. In Fort Worth, North Fort Worth, Arlington, Mansfield and Grapevine special days are devoted to the cleaning up and in many ways rendering the city and towns more healthful and attractive. This work in most instances is being done by the public at large; there is no appropriation or men employed for this special work, except in the city of Fort Worth.

PURE FOOD.

City and county employ Dr. Harold Elderkin at a salary of $100 per month as milk and food inspector. He reports that, with few exceptions, those engaged in this line of business are willing to comply with the legal requirements, and many of them have already made great improvements toward the procurement of more healthful products.

PEST HOUSE AND QUARANTINE.

Including the patients in pest house on January 1, 1908, and those since admitted up to and including May 8, 1908, we have had 198 cases, with one death; outside city and not admitted to pest house we have had 88 cases, there being 21 families infected; in the city of Fort Worth and North Fort Worth cases not admitted to pest house, 110, no deaths. At the present time we have in pest house two cases, one family quarantined in the city of Fort Worth and one in the suburbs. The cost of maintaining these cases for the year ending April 30, 1908, at the pest house was $4955.17. The cost of maintaining those quarantined in the city since January 1, 1908, is approximately $250. County patients, $100. No case of leprosy, scarlet fever, diphtheria, epidemic cerebro-spinal fever, typhoid fever, tuberculosis or trachoma has been reported to me.

Quarantine regulations of smallpox have been complied with in all cases; other contagious troubles are left almost entirely to family physicians, who are instructed with reference to isolation and disinfection. Physicians of county as a rule cheerfully co-operate with local health officers in preventing spread of contagious or infectious diseases. Tuberculosis cases have not as a rule been reported. These cases not having been reported, it is difficult to determine whether or not tuberculosis is on the increase. Commissioners court have given county health officer full authority in management of smallpox cases, but so far have paid no attention to other contagious diseases. The more intelligent of our people believe in the transmissibility of yellow fever by the mosquito and are making a systematic effort to exterminate this dangerous insect. Under conditions mentioned with reference to imported cases of yellow fever, I see no need of quarantine. I think that our people have faith in their State Health Officer's ability and earnestness in his efforts to better the sanitary conditions of the State, and will stand for and aid him in any reasonable matter he may formulate for the protection of public health in this State. I think it unlikely that our small towns would incorporate for the purpose of sanitation only.

### VITAL STATISTICS.

Our doctors now all say they are reporting their births and deaths. Midwives do not report their cases better than physicians.

In county schools vaccination is not required before admittance.

